# Fusion Methods for Face Presentation Attack Detection

**DOI:** 10.3390/s22145196

**Published:** 2022-07-12

**Authors:** Faseela Abdullakutty, Pamela Johnston, Eyad Elyan

**Affiliations:** School of Computing, Robert Gordon University, Aberdeen AB10 7AQ, UK; f.abdullakutty@rgu.ac.uk (F.A.); p.johnston2@rgu.ac.uk (P.J.)

**Keywords:** face presentation attacks, deep learning, feature-fusion

## Abstract

Face presentation attacks (PA) are a serious threat to face recognition (FR) applications. These attacks are easy to execute and difficult to detect. An attack can be carried out simply by presenting a video, photo, or mask to the camera. The literature shows that both modern, pre-trained, deep learning-based methods, and traditional hand-crafted, feature-engineered methods have been effective in detecting PAs. However, the question remains as to whether features learned in existing, deep neural networks sufficiently encompass traditional, low-level features in order to achieve optimal performance on PA detection tasks. In this paper, we present a simple feature-fusion method that integrates features extracted by using pre-trained, deep learning models with more traditional colour and texture features. Extensive experiments clearly show the benefit of enriching the feature space to improve detection rates by using three common public datasets, namely CASIA, Replay Attack, and SiW. This work opens future research to improve face presentation attack detection by exploring new characterizing features and fusion strategies.

## 1. Introduction

Face recognition (FR) is one of the most popular biometric technologies as it is user-friendly, cost-effective, and non-intrusive. Face-presentation attacks (PA) remain a serious concern for the resiliency of FR systems. Imposters gain illegal access bypassing FR systems by using forged facial artefacts, reducing the reliability of these systems. Therefore, face presentation attack detection (FPAD) has been gaining significant attention among the research community [1].

PAs in the form of a photograph, video, or mask of an authorised user can deceive the FR system. These attack images may be reproduced on various media. In addition, video and photo attacks can be displayed on many types of digital devices [2]. A mask attack can be made from different types of materials. Masks can either be flexible, as in silicon masks, or rigid, as in wax masks. Even within a particular type of attack, the spoofing mechanism used will vary [3]. Moreover, technology and social media facilitate the development of novel, sophisticated attacks.

**Figure 1:**
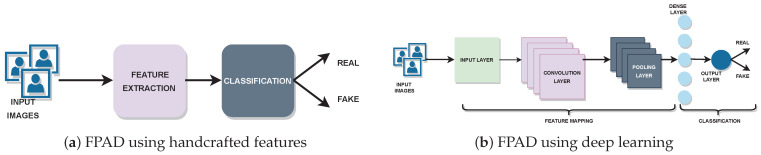
Face presentation attack detection (FPAD) methods: (**a**) The hand-crafted feature method has a feature-extraction module followed by a classifier. Manual feature extraction is carried out in this FPAD method. (**b**) In deep learning-based FPAD, extracted features are passed to the classifier following an end-to-end learning method. Unlike hand-crafted feature methods, CNNs facilitate automatic feature extraction in deep learning based FPAD.

Early FPAD models utilized hand-crafted features such as texture, image quality, and motion, combined with standard classifiers, such as SVM and Random Forest, to determine whether the detected facial image is real or not [4]. Convolutional neural networks (CNN) took the place of these classical feature engineering models. Hand-crafted feature-based FPAD methods are shown in Figure 1a. Figure 1b shows deep learning-based FPAD methods. CNN-based FPAD models benefited from their exceptional inherent feature-extraction capability to some extent. Yet these deep learning-based models failed to reach adequate generalisation against emerging, unseen attacks [5].

There are multiple reasons for the low generalisation capability of FPAD models. The majority of FPAD models were either designed for the detection of specific types of attacks or were trained by using the existing face anti-spoofing (FAS) datasets. However, these FAS datasets have limited variance in size, attack types, and subjects. Moreover, datasets were recorded in a controlled environment that lacked sufficient variation in illumination, recording devices, settings, and the environment [6]. As a result, even if these models detect some specific attack types, they are not reliable in detecting unseen attacks in real-life scenarios. This necessitates the development of more generalised FPAD models to detect PAs [7].

Some recent efforts to improve FPAD have leveraged features from models pre-trained on large datasets designed for object recognition [8,9]. These datasets have high variance across multiple factors. This led to the models performing well in object detection, recognition, and captioning tasks that incorporated deep features from the images. The spoof detection problem does not have large, labelled datasets, unlike these computer vision tasks. Rather, FAS datasets are often collected in controlled environments by using specific types of attacks and a smaller number of subjects. Detecting presentation attacks involves detecting spoof-specific features, such as specular reflection, deformations, glare, spoof patterns, and Moire effects [10]. These features are not always present in high quantities in the common datasets designed for image-classification tasks. Hence, relying on deep models, which were pre-trained on image classification datasets, when the data does not exhibit the necessary features, may not be optimal for improving FPAD performance. Meanwhile, traditional feature extraction methods make use of shallow features. In this approach, the challenge is to select a suitable descriptor that is invariant to factors such as illumination, light, skin type, recording device, and environment. These descriptors should also effectively represent the spoof-specific patterns [5].

PAs, especially 2D attacks, are either printed on different materials or displayed on digital devices [11]. Mask attacks also can be created by printing the genuine face on suitable materials [12]. Such recapturing processes introduce distortions in PAs. The distortions are the cues to distinguish between real and fake faces [13,14]. Texture methods (LBP, HOG, and DOG) were used to extract these cues for PA detection. A good number of texture-based methods used grayscale images, discarding colour feature-related cues. However, colour distortion cues provide significant information for identifying PAs [15,16]. Hence, colour texture analysis was considered in this work to combine with deep features to perform PA detection.

This article proposes and evaluates a fusion method that takes advantage of both handcrafted and deep features. In this study, colour texture features, which provide spoof specific cues, were combined with deep features extracted by using pre-trained image classification models through concatenation. As a result, both the local features and the deep global features were used together as the input to the classifier to determine the authenticity of the facial images. Even though deep feature extraction through transfer learning, colour texture analysis, and their fusion for FPAD are already established methods in the related literature, those methods used traditional machine learning classifiers such as SVM. The fusion method presented in this article takes advantage of neural network based classifiers. Instead of using any of the commonly used pre-trained models, this study compares the fusion method by using three commonly used pre-trained models and a custom CNN model trained from random initialisation. A comparative analysis of computational speed corresponding to baseline and fusion method is also included in this article.

Pre-trained VGG-16, ResNet-50, and Inception V3 and one custom CNN models were used to extract deep features. Before classification, these deep features were combined with the colour texture feature (Colour Local Binary Pattern—CLBP). The baseline models included transfer learning by using VGG-16, ResNet-50, and Inception V3. These pre-trained models were fine-tuned for binary classification by using the FAS datasets CASIA, Replay Attack, and SiW. A custom CNN model was also trained by using the FAS datasets. The CLBP features were combined with deep features from VGG-16, ResNet-50, Inception V3, or the custom CNN model to compare PA detection performance. The key contributions of this are article are:

- A fusion method combining both hand-crafted, colour texture features and deep features from pre-trained CNNs.
- An experimental framework to evaluate face presentation attack detection by using deep CNN models and the fusion models.

The remainder of this article is organised as follows: Section 2 explains the existing literature on fusion strategies for face presentation attack detection. The feature extraction and fusion methods are described in Section 3. Section 4 includes details of the experimental settings and datasets. The results are presented in Section 5 and discussed in Section 6. The paper concludes in Section 7 with possible future directions.

## 2. Related Work

Regardless of the notable advancements in FR systems, they are still vulnerable to PAs. Emerging new PA variants also pose a serious problem to the generalisability of existing FPAD models [7]. Consequently, the research community has been investigating various techniques to improve generalisation in FPAD by using distinct techniques.

Local binary patterns (LBP) and its variants have been extensively used in handcrafted feature methods for FPAD. The authors of [3] proposed a novel fusion model to reduce training parameters by using the similarities between CNNs and LBP extraction. This fusion network reduced the number of network parameters by using a statistical histogram. Nevertheless, the model failed to detect some specific types of attacks. Chen et al. [17] fused colour texture features with deep features from the images. Colour texture features were extracted by using rotation invariant local binary pattern (RI-LBP). These location features were fused with the global features extracted by using a ResNet model for classification. An SVM, with RBF kernel, classified these fused features to detect whether the face was authentic or spoofed. The experiments considered YCbCr and HSV colour spaces. In a comparison of grayscale, RGB, YCbCr, and HSV, the texture features, combined from the YCbCr and HSV colour spaces provided better detection results. The authors also presented a cross-dataset evaluation to show the generalisation capability of the method. The authors of [18] combined different handcrafted features including LBP, GDP, GLTP, LDIP, LGBPHS, and LPQ. These extracted features were classified by using the K-NN classifier. However, the model exhibited very low real-face detection accuracy regardless of the high (98.39%) fake-face detection accuracy. Moreover, this method only combined handcrafted features. Deep global features were not considered in this model.

Liu et al. [19] adopted a multi-modal data fusion strategy to identify fake faces. The model combined both low-level and high-level features from RGB and IR images for FPAD. This model exhibited generalisation against different conditions such as dim light, realistic face camouflage, static or motion pattern, etc. Because a nonlinear fusion method was used with multi-modal data, the generalisation was enhanced to some extend. The authors of [20] followed a dual cue fusion method to mitigate the error in FPAD. The framework had two streams. The first stream used facial images with background and the second stream used face images after facial area only. Fast Fourier Transform (FFT) was extracted from the facial images with background and these images were used to train CNN model for FPAD. Simultaneously, the second stream carried out a colour space (HSV) transformation on facial area RGB images. Texture features were extracted from these images as an input to SVM classifiers for FPAD. The decisions from both streams were combined to identify the PAs.

Younis and Abuhammad [21] proposed a hybrid fusion framework to address PAs by using multiple biometric modalities. The authors combined transfer learning and hand-crafted feature methods by using discriminant correlation analysis (DCA) and canonical correlation analysis (CCA). On the images, contrast adjustment was carried out to control intensity distribution. Histogram of gradient (HoG) features were extracted from these images. A multi-level fusion strategy was followed to incorporate multiple biometric modalities. In a dual stream fusion model, Fang et al. [22] used frequency domain features and complementary RGB features. This model included a hierarchical attention module as well as a multi-stage fusion strategy. A special attention module at the lower layers of CNN enabled extraction of texture features. Similarly, a channel attention module at the higher layers extracted deep semantic features. Because both of these features are essential for better detection of attacks, this fusion model addressed generalisation through decomposition of multi-level frequency.

Unlike other fusion models existing in the literature, the authors of [23] combined deep learning with serial fusion, as parallel fusion models have a longer response time. This multiple biometric modalities-based method used Siamese neural networks. Deep networks were used for deep feature extraction and match score generation. Daniel and Anitha [24] proposed a new FPAD method, combining texture and image quality features. The image colour space was changed to HSV. Entropy-based colour texture features and image quality features were extracted from these HSV images. Later, these extracted features were concatenated and then classified. Even though this model combined different handcrafted features, it did not consider deep feature extraction to address PAs.

Xu et al. [25] used two lightweight networks to learn motion and texture cues in order to improve PAD. An element-wise weighing fusion strategy was followed in this model. In [26], the authors used camera-invariant feature learning while focusing on generalisation in FPAD. This framework learned both high-frequency and low-frequency information. A module in the framework carried out high-frequency domain camera-invariant feature decomposition. Another module in the framework performed image re-composition of both high- and low-level information. Classification results of both modules were fused together by using a weighting strategy to perform final classification. Sharifi [27] proposed a decision-level fusion strategy to address FPAD. The author carried out feature extraction with a Log–Gabor filter. By using a nearest neighbors classifier, the scores were classified. Simultaneously, feature extraction and classification was performed by using a CNN model too. By using the OR rule, the decisions from the two modules were fused to get a final decision on the genuineness of the facial image.

Cai et al. [28] used metapattern learning, instead of hand-crafted feature extraction, to create a hybrid model to address FPAD. By using the hierarchical fusion module (HFM), RGB image and metapatterns were combined and passed to a CNN for further classification. Song et al. [29] proposed FPAD by using least squares weight fusion (LSWF) of channel-based feature classifiers. The authors utilised colour, texture, spatial domain, and frequency domain features extracted from different channel spaces along with convolutional features in this fusion method. To assign the optimal weights of classification score fusion, a least square weight fusion strategy was used.

Anand and Viswakarma [30] proposed a fusion method combining deep features and colour texture features. Extracted features were classified by using SVM separately. The probabilities from each model were fused to get the final probability. The authors of [31] utilised dynamic texture features and shape cues in a fusion method, to address 3D attacks. Geometric information used in this method were either extracted by the depth sensors or reconstructed from the RGB images. It also made use of multi-modal dynamic fusion network and 3D model-guided data augmentation. This data augmentation facilitated data in different poses, which in turn assisted in training the network fully.

## 3. Method

An experimental framework is used to detect PAs by fusing deep and hand-crafted features. For the evaluation, three publicly available datasets, CASIA [2], Replay Attack [32] and SiW [33] were used. For this fusion method, texture was extracted from the images by using colour texture analysis (CLBP) [16]. By using pre-trained deep learning models, VGG-16 [34], ResNet-50 [35], and Inception V3 [36], deep features were extracted. These high level features from deep models and low level features from colour texture analysis were then concatenated and passed to the classifier. The classifier consisted of a dense layer with 512 units and a sigmoid layer. Additionally, a custom CNN model was trained only on each dataset individually in order to compare with the pre-trained networks. The resultant features were combined with the colour texture features and passed to a classifier as before. The fusion method used deep features from pre-trained and custom CNN models in different evaluation scenarios to compare the impact of fine-tuning and fully training models on FAS datasets.

The experiments also consisted of baseline methods. The pre-trained and custom CNN models were trained for binary classification. As in the fusion methods, the baseline classifier also consisted of a dense layer with 512 units size and sigmoid layer. All the evaluation scenarios used binary cross entropy as the loss function and Adam as the optimiser.

### 3.1. Pre-Trained Models

FPAD is conventionally treated as a binary image-classification problem. Hence, FPAD also takes the advantage of transfer learning to address dataset limitation. VGG-16 [8] and ResNet-50 [37] have been used previously to address FPAD by using transfer learning. Lucena et al. [8] fine tuned the VGG-16 model by changing the top layers for detecting PAs by using binary classification. Nagpal and Dubey [9] used Inception-V3 and ResNet-50 models for the PA detection. According to the authors, transfer learning with these pre-trained models facilitated better detection performance than training from a random weight initialisation. The authors of [37] also used ResNet-50 for the FPAD task.

Pre-trained models, VGG-16, ResNet-50, and Inception V3 were used for binary classification as well as feature extraction in the presented experimental framework in this work. These deep network models were pre-trained for image classification [38]. The features were extracted by removing the output layer from the models. VGG-16 feature vector size was 4096. Feature vectors of size 2098 were extracted from ResNet-50 and Inception V3. For binary classification, the top layers were replaced with a fully connected layer of size 512 and sigmoid layer in these pre-trained models. Thus, transfer learning was applied in the baseline methods.

### 3.2. Convolutional Neural Network (CNN) Model

**Figure 2:**
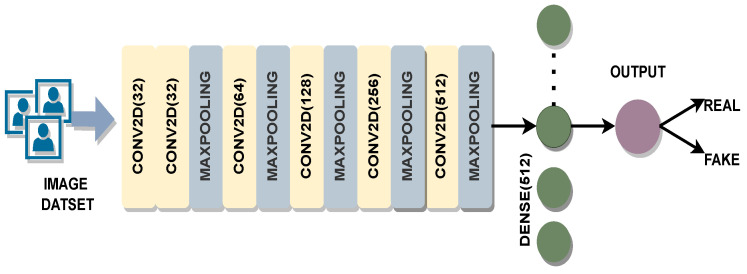
Custom CNN Model: A custom CNN model was trained on CASIA, Replay Attack and SiW training sets for the evaluation using each respective test set.

Evaluation was also performed by using a custom CNN model. The model has five convolution layers, each followed by a max-pooling layer. The classification block in this model is formed by using a dense layer of size 512 and a sigmoid layer (Figure 2). From block 1 to block 5, the number of filters varied from 32 to 512. A kernel size of 3 × 3 was used in each convolutional layer. The models were trained by using a corresponding FAS dataset used in the experiments. The weights from these models were also used for feature extraction in the fusion method.

Similar to pre-trained models, the custom CNN model was also used to extract deep features by removing the output layer. This provided a feature vector of size 512. Compared to the deep models, VGG-16, ResNet-50, and Inception-V3, this CNN architecture was shallow, with only 8 layers. The CNN model used for feature extraction was shallower compared to the pre-trained models.

### 3.3. Colour Texture Analysis

Presentation attacks include photos printed on different mediums, video or photo displayed on digital devices, and masks. The spoofing medium varies in resolution and display quality. Grayscale image-based texture analysis facilitates identification of high-quality PA. The grayscale-based methods (e.g., LBP) cannot provide sufficient difference in textural cues when the quality of the PAs diminishes [15]. A PA image or video passes through at least two cameras and a printing or display medium. Hence, many PAs have a recapturing effect. Compared to an authentic capture, the colour reproduction of these spoofing mediums would be limited. Hence, PA will have the colour features corresponding to the printing or displaying medium gamut. Moreover, the recapturing camera and the entire recapturing process to perform PA can cause colour disparities and imperfections.

Human eyes are more sensitive to luminance than chrominance. Hence, the colour reproduction mapping in printing or display process preserve luminance variation in the source image rather than chrominance. Thus, the PAs may contain chrominance variations which are largely invisible to human vision. These chrominance variation cues can be utilised to distinguish between real and fake facial images. The majority of the available FAS datasets provide RGB images or videos. On the other hand, RGB colour space has high correlation between the colour components. The RGB colour space does not adequately separate luminance and chrominance information. Recapturing introduces chrominance variation in PAs, while sustaining luminance variation. It is unlikely that RGB colour space would be able to determine spoof-specific chrominance cues. Thus, alternative colour spaces should be used to extract such discriminatory cues [16].

**Figure 3:**
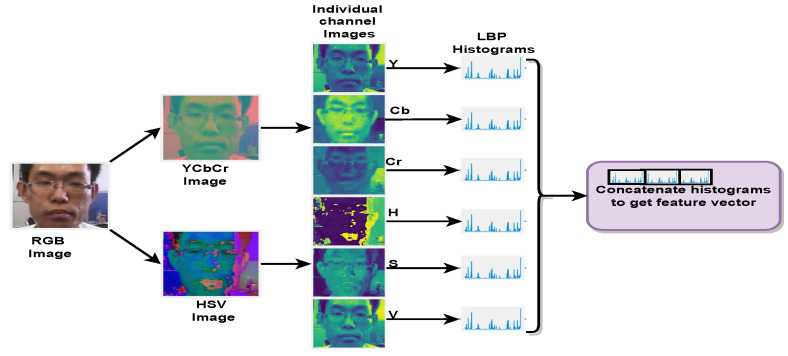
Colour texture analysis: RGB images were converted to YCbCr and HSV colour spaces. LBP histograms for each channel in these colour spaces were calculated individually. These 6 histograms were concatenated to obtain a final feature vector of size 354.

By analyzing the chroma channel colour texture, the local colour disparities discussed above can be identified. Luminance and chrominance are represented in YCbCr colour space. The chrominance component of YCbCr reveals disparities which are presented in PAs. HSV colour space represents hue, saturation, and brightness. HSV colour space contains a chrominance component, which is complementary to that in YCbCr colour space. Both of these color spaces provide chrominance components that can be used to identify PA. Hence, colour texture analysis was used to extract the hand-crafted feature in this proposed fusion method. Although deep networks provide global features, extracted features include the local (chroma) cues. Colour texture analysis [16] addresses these variations, extracting LBP from individual channels from the images. Hence, RGB images were converted to HSV and YCbCr colour spaces to extract related features to identify PAs. Figure 3 presents the process of colour texture analysis. To extract the colour texture features, the channel-wise components were separated after conversion to each colour space. An LBP histogram for each channel was calculated. The histograms from these 6 channels (H, S, and V in HSV colour space and Y, Cb, Cr in YCrCb colour space) were then combined to form the final feature vector of size 354.

### 3.4. Fusion Method

The experimental framework extracted and combined high-level deep features and lo- level local color texture features. Deep features were extracted by using transfer learning and custom CNN models. VGG-16, ResNet-50, and Inception V3 were used to get features’ vectors of size 4096, 2048, and 2048 respectively. As mentioned in Section 3.1, these models were pre-trained on ImageNet. On the other hand, custom CNNs were trained on three FAS datasets and used to extract feature vectors of size 512. Because the RGB image was converted into HSV and YCbCr colour spaces, there were six channels in total. As the texture feature extraction using LBP was carried out on each channel, these channels provide a feature vector of length 59. Thus feature vectors from these six channels forms a low-level feature vector of size 354.

Concatenation is an effective way to combine different features for use in machine learning. Extracted CLBP features were concatenated with features either from pre-trained models or a custom CNN model. Thus, by concatenating sets of deep and handcrafted features, a final feature vector was created. Let $F^{Deep}$ be the deep feature vector with size *m* and $F^{CLBP}$ be the colour texture feature vector with size *n*. Then the final feature vector $F^{Fusion}$ can be represented [39] as

$$F^{Fusion}=F^{Deep}\cup F^{CLBP}.$$

Thus $F^{Fusion}$ will have the size (m+n). Here $F^{CLBP}$ had size 354. *m*, the size of $F^{Deep}$ feature vector, held different values according to the pre-trained or custom CNN models, which was used for feature extraction. Hence the size of $F^{Fusion}$, *m* was determined based on the deep model used for feature extraction.

**Figure 4:**
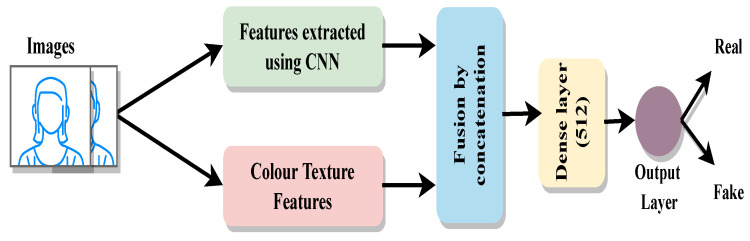
Fusion Method: In total, 4 different models were built, one for each of the pre-trained deep neural networks plus a custom CNN trained on each respective dataset. In each model, the extracted deep features were concatenated with colour texture features and then passed to the classifier for PA detection. The classifier consisted of a fully connected layer of size 512 and a sigmoid layer.

Figure 4 illustrates the structure of the proposed framework. It consisted of a deep feature extraction module and a hand-crafted feature extraction module. The resultant feature vectors from these modules were the input of the fusion module. Fusion simply involved concatenation. This combined feature vector was then passed to the classifier. The classifier consisted of a dense layer of size 512 followed by a sigmoid. However, the input size of the classifier was different according of the different feature vector size.

## 4. Experiment

This experimental framework evaluated baseline models and proposed a fusion method using three FAS datasets, namely CASIA, Replay Attack, and SiW. The pre-trained models were fine-tuned to carry out PA detection. The customised model was trained by using the aforementioned three FAS datasets. The datasets and experimental settings including hyper-parameter tuning are explained below.

### 4.1. Datasets

**Figure 5:**
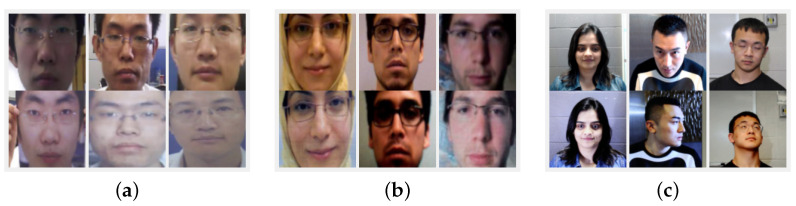
Real and fake facial image samples from (**a**) CASIA, (**b**) Replay Attack, and (**c**) SiW. Upper row in each figure contains the real-face samples, whereas the lower row has the PA samples.

By using three widely used public FAS datasets, CASIA, Replay Attack, and SiW, the proposed fusion method was evaluated. These FAS datasets mainly include print and replay attack PA types. The datasets differ from each other in terms of size, gender ratio, ethnicity, recording devices, spoofing medium, illumination, and settings. Figure 5 shows both real- and fake-face samples from three datasets. The top row in Figure 5a–c has genuine facial images. The lower row shows the corresponding fake facial images.

CASIA has fake- and real-face videos from 50 subjects. The dataset contains photo attack variants including print, warped photo, and cut photo. Video attacks are also part of this public dataset. Corresponding to each subject, there are 3 real-face and 9 fake-face videos. Thus a total of 600 videos are included in the CASIA dataset. The training set is made of videos of 20 subjects. The remaining videos from 30 subjects are included in the test set. The train and test sets are disjointed in terms of subjects. CASIA has videos of three qualities; low, normal, and high. This dataset was recorded in natural scenes. This process did not use any kind of artificial unification while recording the dataset. Because cut photos attacks were included in CASIA, eye blinking was also incorporated in the videos. Print attacks with better quality were reproduced by using copper papers. However, CASIA includes only subjects from a single ethnicity, i.e., Asian. Even though 10% of the subjects were females, the training partition is devoid of female subjects. Hence there is no gender variance in the training partition.

In the Replay Attack dataset, videos from 50 subjects are distributed among training, development, and test sets. The training and development sets have 15 subjects each, whereas the test set has 20 subjects. Fake faces were displayed as printed photos, on mobile and tablet displays. During the recording process, these three mediums were either fixed or held by the operator. Both controlled and uncontrolled settings were used. Uniform background with illumination using incandescent lamps was used in controlled settings. An uncontrolled setting made use of non-uniform background and natural light for illumination. The Replay Attack dataset also had a female-to-male subject ratio 1:9. However, unlike CASIA, this dataset has both gender in all the data partitions. This dataset also has variance in terms of ethnicity.

SiW is the third dataset used for the evaluation of fusion method. Compared to the other two public datasets used for experiments, the SiW dataset includes variance in terms of ethnicity, poses, illuminations, expressions, and distance-to-camera. A total of 4620 videos from 165 subjects include 8 real face and 20 attack videos corresponding to each subject. A total of 27% of the subjects in SiW datasets are females. It has subjects belonging to different ethnicity: 35% Asian, 35% Caucasian, 7% African American, and 23% Indian people are included in the dataset, giving much more variance in ethnicity. Among the subjects, 44% have glasses and 20% have beards. Unlike the CASIA and Replay Attack datasets, which have only a frontal pose range, SiW has pose ranges of [−90,90]. Moreover, SiW used artificial illumination. Table 1 shows a comparison of the three datasets in different aspects.

### 4.2. Experimental Settings

**Figure 6:**
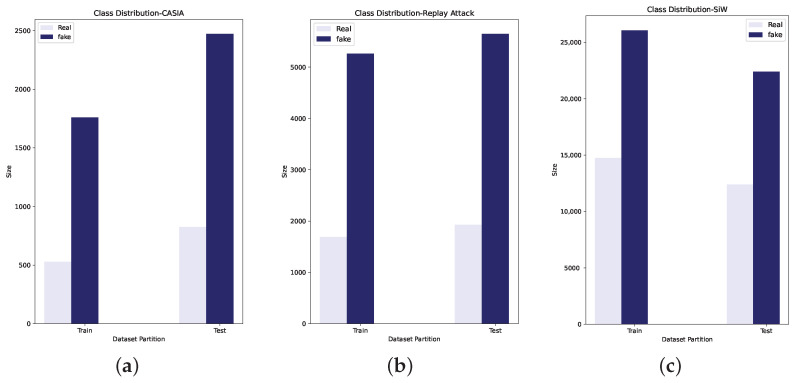
Test and train class distribution after image extraction from CASIA, Replay Attack, and Spoof in the Wild. (**a**) CASIA, (**b**) Replay Attack, (**c**) SiW.

Three FAS datasets, CASIA, Replay Attack, and SiW were used to evaluate the fusion method. These datasets are available in video format. Frames from CASIA and Replay Attack were extracted at a rate of 2 fps and faces from these frames were detected. For the SiW dataset, frames were extracted at 1 fps and, by using given annotations, face detection was carried out. In addition, some random scaling of the bounding box for SiW was performed in order to provide some background information and improve facial image diversity. Facial images from three datasets were resized to 224 × 224 pixels. Rather than using customised data partitions to address generalisation [1], the official train-test split was maintained. Table 2 shows the number of training and test images in each dataset. Figure 6 shows the data distribution corresponding to each dataset.

Intra-dataset evaluation was conducted for the baseline and fusion methods. Images of 224×224 size were used in the evaluation. In the baseline method, the training used 10 epochs and 32 batch size with CASIA and Replay Attack. Training with SiW was carried out with a batch size of 512. The Adam optimiser [40] with different learning rates were used in the experiments. VGG-16 as well as ResNet-50 used a learning rate $10^{-5}$ whereas Inception V3 used $10^{-6}$. A customised CNN model used different learning rates while training with different datasets. Learning rates of $10^{-6}$, $10^{-5}$, and $10^{-4}$ were used while training with CASIA, Replay Attack, and SiW respectively. The Python programming language was used for implementing experiments by using Keras with TensorFlow on the backend. Experiments were conducted on NVIDIA DGX-1 machine, using a single GPU system.

The results are reported in terms of accuracy, half total error rate (*HTER*), precision, recall, F1 score, false positive rate (*FPR*) and false negative rate (*FNR*). *HTER* is the average of *FPR* and *FNR*. The equations to calculate *FPR*, *FNR* and *HTER* are given below.

$$FPR=\frac{FP}{FP+TN}$$

$$FNR=\frac{FN}{FN+TP}$$

$$HTER=\frac{FNR+FPR}{2}$$

where *FP* is false positive, *TN* is true negative, *FN* is false negative, and *TP* is true positive.

## 5. Results

Fusion and corresponding baseline methods were evaluated by using CASIA, Replay Attack, and SiW datasets. For deep feature extraction, pre-trained models, VGG-16, ResNet-50, and Inception V3 were used. Custom CNN models trained on the three FAS datasets were also used for deep feature extraction. The fusion method combined deep features from each model with CLBP features and then classified by using a neural network-based classifier. This classifier had an input layer, one dense layer of size 512 units, and a sigmoid layer. Evaluations were carried out to compare PA detection accuracy, HTER, and computational speed for both baseline and fusion methods.

**Figure 7:**
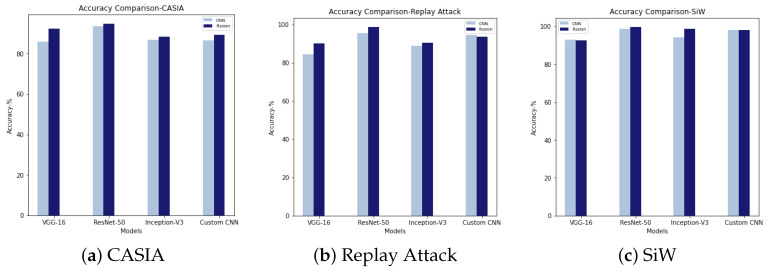
Accuracy comparison for CASIA, Replay Attack, and SiW.

**Figure 8:**
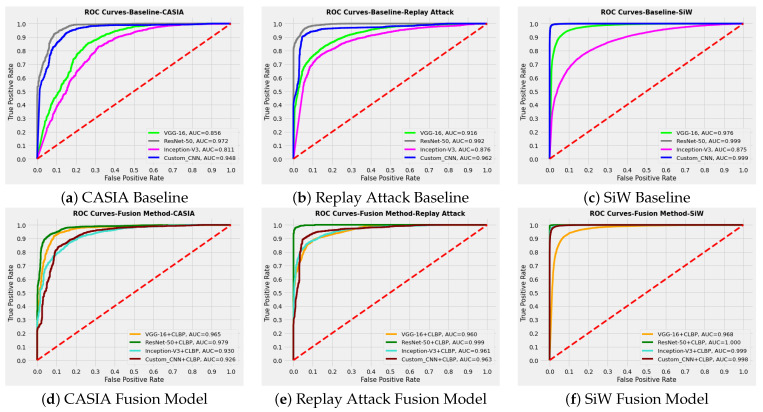
ROC curve analysis for CASIA, Replay Attack, and SiW. The performance of FPAD using pre-trained and custom CNN models is shown in (**a**–**c**). Fusion method performance for respective datasets is presented in (**d**–**f**).

PA detection performance of baseline models are presented in Table 3. Binary classification using pre-trained and custom CNN models were considered as the baseline methods. Table 4 represents the results of fusion methods. A graphic representation of accuracy comparison is also presented in Figure 7. Figure 8 shows the ROC curve corresponding to baseline and fusion models.

Transfer learning using ResNet-50 had the highest accuracy among the baseline models for CASIA (93.36%), Replay Attack (95.57%), and SiW (98.78%). A custom CNN model performed better than VGG-16 and Inception V3 with Replay Attack, and SiW. However, with CASIA, all three pre-trained models had better detection rate than the custom CNN model in baseline evaluation (Table 3). However, ResNet-50 (98.78%) and custom CNN (98.16%) models exhibited very close detection accuracy in baseline evaluation with SiW.

From the fusion method results in Table 4, it is evident that the detection improved for almost all the datasets, regardless of the models used for deep feature extraction. Thus fusing colour texture features with deep features improved PA detection in intra-dataset evaluation. Among the models used, the combination of colour LBP (CLBP) with ResNet-50 features showed the best performance (Table 4).

A graphic comparison of detection performance of pre-trained and custom models is shown in Figure 7. It shows that fusing CBLP features with CNN-extracted features largely improves detection performance across the board. The main exception is the model using a custom CNN to extract the features. For Replay Attack, the detection performance was slightly reduced when the deep feature extraction was carried out by using the customised CNN model. However, with pre-trained models, the Replay Attack dataset also improved PAD performance. In the evaluation with SiW, both VGG-16 + CLBP and customised CNN + CLBP exhibited hardly any improvement. Nevertheless, ResNet-50 + CLBP and Inception V3 + CLBP improved compared to networks without CBLP. Comparing both Table 3 and Table 4, it can be seen that FPR and FNR were reduced in the proposed method compared to the baseline method. The decreased FPR and FNR resulted in a lower HTER than the baseline in fusion methods, which in turn improved PA detection.

Figure 8 shows the ROC curve analysis corresponding to three datasets for baseline and fusion models. In the baseline method, which uses binary classification using CNN, the best performance was exhibited by the ResNet-50 pre-trained model. The customised CNN model also showed a very close performance to ResNet-50 in the baseline method. However, this CNN model performed better than VGG-16 and Inception-V3 with all three datasets despite having far fewer layers. Among the fusion models, the combination of colour LBP with ResNet-50 features provided the highest detection performance. With CASIA, the customised CNN model features led to a performance very close to Inception-V3, but lower than ResNet-50 and VGG-16. However, with Replay Attack and SiW, this model features performed even better than VGG-16 and close to ResNet-50 in combination with CLBP.

Table 5 presents a comparison between the computational speed of the baseline and fusion methods for each dataset. Computational speed decreased substantially with VGG-16 and custom CNN model features in the fusion method. Training times of the fusion models reduced to a value of less than 50% of the baseline training time for these two models, with three datasets. At the same time, the PA detection accuracy increased by a value of 6% for CASIA and Replay Attack when VGG-16 features were combined with CLBP features. SiW showed slightly lower accuracy in fusion method with VGG-16 deep features. Even though the custom CNN model-based evaluation scenario had improved computational speed in the fusion method, it did not facilitate improvement in PA detection compared to the corresponding baseline model for Replay Attack and SiW. Only CASIA showed better accuracy when combining the shallow features with the custom CNN. For ResNet-50 and Inception V3, computation speed deteriorated in fusion methods, regardless of the improved accuracy. The times taken for training ResNet-50 and Inception V3-based fusion models were much higher than the corresponding baseline values. However, in ResNet-50-based evaluation scenarios, the largest dataset, SiW, exhibited slightly better accuracy and computational speed in the fusion method than the baseline. The SiW/ResNet-50 combination shows that the fusion method has a slightly faster training time and this implies that, with even larger datasets, the fusion method might have some advantages over the baseline method in both speed and accuracy. However, it should be noted that the fusion feature extraction method has not been fully optimised in these experiments, and it might be possible to improve fusion method training times with some simple software optimisation.

## 6. Discussion

Presentation attack detection performance exhibited an overall improvement by using the proposed fusion method. Combining local texture features, extracted from different channels with deep features was largely effective in reducing the error in identifying real faces from fake faces. This caused the increment in accuracy and reduction in FPR and FNR.

For CASIA, the fusion method with pre-trained models, reduced FPR, and increased FNR. However, customised CNN, which was trained by using the FAS dataset shows the opposite behaviour with CASIA. FNR deceased and FPR increased. Consequently, features extracted by using pre-trained models as well as the customised CNN model in combination with hand-crafted features provided improvement in PA detection when evaluated with CASIA. A similar performance was given by the Replay Attack dataset. SiW showed a different performance to that of CASIA and Replay Attack because the SiW dataset is “in the wild”. Hence, it might not exhibit dataset biases that can be easily exploited by networks trained only on that dataset (i.e., the custom CNN). The other two datasets were recorded under more controlled settings. That is why the results indicated a higher level of capture bias in CASIA and Replay Attack datasets. ResNet-50 and Inception V3 models with colour texture analysis substantially decreased FPR and FNR when evaluated with this dataset. However, for VGG-16 and customised CNN, the FPR and FNR increased slightly, reducing the performance in fusion method.

The custom CNN models used in the experiment were trained and tested by using corresponding FAS datasets. In fact, the features extracted by using this model, when concatenated with CLBP features, in general increased either or both of FPR and FNR. This clearly shows that the model needs further tuning to improve the PA detection performance. The performance analysis also shows that feature extraction by using ResNet-50 is most effective for FPAD among the considered pre-trained models. The fusion models also illustrate the suitability of colour texture analysis in this strategy.

From the ROC analysis curves, it is evident that CNN model trained with the corresponding dataset performed very close to or better than the deep networks considered in the experiments. ResNet-50, Inception-V3, and VGG-16 have 50, 48, and 16 layers, respectively. The customised CNN model has 13 layers, making it shallower than the other models. However, these deep models were trained on the ImageNet dataset, whereas each custom CNN model was trained on the respective FAS dataset training set. This implies that a small dataset and shallower network can achieve comparable or better performance than deep, pre-trained networks.

The computational speed presented in Table 5 included the time required to extract deep features and hand-crafted features, and train the classifier model. For each dataset, the hand-crafted feature extraction time is the same. Moreover, the classifier training period is significantly less than the time taken for feature extraction. The variation in the recorded computational speeds relies upon deep feature extraction speed. Hence, a pre-trained model with depth equal to or less than VGG-16 could be used to extract deep features to improve the performance of this fusion method by using CLBP. From the results, it is also evident that the performance of shallow models trained on FAS datasets was not improved by fusion. This implies that the features that emerge in these shallow models may already encompass the shallow, engineered features. Other suitable hand-crafted features could also be combined with the custom CNN models trained on FAS datasets to investigate the impact of custom CNN model deep features. A challenge is extracting suitable handcrafted features which can provide spoof-specific patterns and further increase FPAD performance, specifically in unseen attack detection.

The comparative analysis using accuracy, HTER, and computational speed which are presented in Table 3, Table 4 and Table 5 points to the advantages, drawbacks, and challenges of the proposed fusion method. The fusion method performed better in PA detection when deep features were extracted by using pre-trained models than the models which were trained with FAS datasets. Among the pre-trained models considered for evaluation, the model with the fewest layers (VGG-16) showed improvement in computational speed and detection performance. One possible hypothesis for this is that FPAD relies on low-level, spoof-specific features, rather than complex deep features. Deeper pre-trained models were able to improve detection performance at the cost of computational speed. However, for application in real-life scenarios, the best model would exhibit optimal performance both in accuracy and computational speed.

## 7. Conclusions

An experimental framework combining hand-crafted and deep features was presented in this article. For hand-crafted features, colour LBP was extracted after converting RGB images to YCbCr and HSV colour spaces. For deep features, both pre-trained models and a custom CNN model were considered. The proposed method was compared to the binary classification with deep learning models. A comparison between these two models showed that hand-crafted features, when combined with the deep features, substantially reduced the number of false positives in most cases. This indicated that rather than deep features, spoof-specific features would facilitate better PA detection in FR systems. Moreover, a fusion method using pre-trained deep models also improves computation speed on some models and shows promise for experiments with larger datasets.

This experimental framework can be further extended in multiple ways. For future work, cross-dataset evaluation may be explored to confirm whether these relatively shallow features exhibit good, generalisable qualities. Alternatively, the fusion method may be expanded by using more features corresponding to texture, frequency, and image quality to detect PAs.

## Figures and Tables

**Table 1:** Comparison of FAS datasets used in the evaluation of fusion method.

Dataset	Subjects	Live Videos	Attack Videos	Attack Types	Display Devices
CASIA	50	150	450	2 Print, Replay	iPad
Replay Attack	50	200	1000	Print, 2 Replay	iPhone 3GS, iPad
SiW	165	1320	3300	2 print, 4 Replay	iPad Pro, iPhone 7, Galaxy S8, Asus MB168B

**Table 2:** Datasets used in experiments and their sample size in train and test partitions.

Dataset	CASIA		Replay Attack		SiW	
Class	Train	Test	Train	Test	Train	Test
Real	527	824	1689	1,928	14,733	12,390
Fake	1760	2471	5261	5645	26,057	5645
Total	2287	3295	6950	7573	40,790	34,779

**Table 3:** FPAD results using deep CNN models.

Dataset	Model	ACC (%)	HTER (%)	Precision	Recall	F1score	FNR (%)	FPR (%)
**CASIA**	VGG-16	85.85	24.01	0.87	0.96	0.91	4.29	43.69
	ResNet-50	**93.36**	**12.60**	0.92	0.99	0.96	0.69	24.51
	Inception V3	86.74	24.39	0.86	0.98	0.92	2.10	46.60
	Custom CNN	86.42	14.84	0.94	0.88	0.90	12.34	17.35
**Replay Attack**	VGG-16	84.25	23.05	0.88	0.92	0.90	8.18	37.91
	ResNet-50	**95.57**	**8.07**	0.95	0.99	0.97	0.66	15.51
	Inception V3	88.78	19.94	0.88	0.98	0.93	2.18	37.71
	Custom CNN	94.39	6.75	0.97	0.96	0.96	4.43	9.23
**SiW**	VGG-16	93.02	7.92	0.94	0.95	0.95	4.33	11.04
	ResNet-50	**98.78**	**1.57**	0.98	1	0.99	0.38	2.75
	Inception V3	94.35	6.53	0.95	0.97	0.96	3.48	9.57
	Custom CNN	98.16	2.24	0.98	0.99	0.99	0.85	3.63

**Table 4:** Fusion methods results.

Dataset	Model	ACC (%)	HTER (%)	Precision	Recall	F1score	FNR (%)	FPR (%)
**CASIA**	VGG-16 + CLBP	92.33	13.26	0.92	0.98	0.97	2.06	24.39
	ResNet-50 + CLBP	**94.65**	**8.68**	0.95	0.98	0.96	1.74	15.29
	Inception V3 + CLBP	88.31	18.54	0.90	0.95	0.92	4.82	32.28
	Custom CNN + CLBP	89.34	15.47	0.92	0.94	0.93	5.87	25.12
**Replay Attack**	VGG-16 + CLBP	90.18	15.14	0.92	0.96	0.94	4.30	25.99
	ResNet-50 + CLBP	**98.56**	**2.64**	0.98	1.00	0.99	0.19	5.08
	Inception V3 + CLBP	90.38	16.19	0.91	0.97	0.94	2.82	29.56
	Custom CNN + CLBP	93.64	8.51	0.96	0.96	0.96	4.13	12.91
**SiW**	VGG-16 + CLBP	92.65	8.47	0.93	0.95	0.94	4.59	12.34
	ResNet-50 + CLBP	**99.60**	**0.48**	1.00	1.00	1.00	0.20	0.76
	Inception V3 + CLBP	98.61	1.57	0.99	0.99	0.99	0.95	2.20
	Custom CNN + CLBP	97.96	2.42	0.98	0.99	0.98	1.13	3.70

**Table 5:** Computation speed V/s accuracy comparison between baseline and fusion methods (training times).

Dataset	Model	Baseline		Fusion Method	
		Computational Speed (s)	Accuracy (%)	Computational Speed (s)	Accuracy (%)
**CASIA**	VGG-16	**785.86**	85.85	**339.76**	92.33
	ResNet-50	545.66	93.36	746.637	94.65
	Inception V3	229.47	86.74	457.27	88.31
	Custom CNN	2166.59	86.42	408.91	89.34
**Replay** **Attack**	VGG-16	**2388.57**	84.25	**945.32**	90.18
	ResNet-50	1611.92	95.57	2261.05	98.56
	Inception V3	663.67	88.78	1366.84	90.38
	Custom CNN	6643.48	94.39	1228.12	93.64
**SiW**	VGG-16	**16,045.91**	93.02	**5468.71**	92.65
	ResNet-50	14,703.66	98.78	13,382.73	99.60
	Inception V3	5711.11	94.35	7979.04	98.61
	Custom CNN	34,430.00	98.16	7185.88	97.96

## Data Availability

The experiments in this research article used three public Face Anti-Spoofing datasets. They are They are CASIA [32], Replay Attack [2], and SiW [33].
